# Extracellular Polymeric Substance Production and Aggregated Bacteria Colonization Influence the Competition of Microbes in Biofilms

**DOI:** 10.3389/fmicb.2017.01865

**Published:** 2017-09-27

**Authors:** Pahala G. Jayathilake, Saikat Jana, Steve Rushton, David Swailes, Ben Bridgens, Tom Curtis, Jinju Chen

**Affiliations:** ^1^School of Engineering, Newcastle University, Newcastle upon Tyne, United Kingdom; ^2^School of Natural and Environmental Sciences, Newcastle University, Newcastle upon Tyne, United Kingdom; ^3^School of Mathematics, Statistics and Physics, Newcastle University, Newcastle upon Tyne, United Kingdom; ^4^Centre for Synthetic Biology and the Bioeconomy, Newcastle University, Newcastle upon Tyne, United Kingdom

**Keywords:** individual-based model, biofilm, competition, EPS, aggregates, quorum sensing

## Abstract

The production of extracellular polymeric substance (EPS) is important for the survival of biofilms. However, EPS production is costly for bacteria and the bacterial strains that produce EPS (EPS+) grow in the same environment as non-producers (EPS−) leading to competition between these strains for nutrients and space. The outcome of this competition is likely to be dependent on factors such as initial attachment, EPS production rate, ambient nutrient levels and quorum sensing. We use an Individual-based Model (IbM) to study the competition between EPS+ and EPS− strains by varying the nature of initial colonizers which can either be in the form of single cells or multicellular aggregates. The microbes with EPS+ characteristics obtain a competitive advantage if they initially colonize the surface as smaller aggregates and are widely spread-out between the cells of EPS−, when both are deposited on the substratum. Furthermore, the results show that quorum sensing-regulated EPS production may significantly reduce the fitness of EPS producers when they initially deposit as aggregates. The results provide insights into how the distribution of bacterial aggregates during initial colonization could be a deciding factor in the competition among different strains in biofilms.

## Introduction

Biofilms are surface associated communities of bacteria that are surrounded by adhesive extracellular polymeric substance (EPS) (Davey and O'toole, 2000) which not only provides them with mechanical integrity but also allows resistance against attack from foreign entities. Understanding the dynamics of growth and competition between several microbial species in a biofilm is crucial for our understanding of chronic diseases such as cystic fibrosis, infection in medical devices, biofouling and various processes used in wastewater treatment. Mathematical models such as Cellular Automaton (CA) and Individual-based Models (IbMs) (Kreft et al., 2001; Picioreanu et al., 2004; Xavier et al., 2005; Nadell et al., 2008; Lardon et al., 2011; Jayathilake et al., 2017) have been instrumental in providing insights into the spatiotemporal growth and competition of microbes under varying conditions. Kreft et al. (1998) proposed the use of IbM as a bottom-up approach which attempts to predict community behavior based on the actions and characteristics of the constituent individuals. The IbM was introduced to cope with artifacts which occurred due to the discrete displacement of biomass in CA (Picioreanu et al., 2004; Tang and Valocchi, 2013). As Ib modeling leads to more realistic biofilm structures (Kreft et al., 2001), it has been widely used to study social evolution in biofilms (Kreft, 2004; Xavier and Foster, 2007; Nadell et al., 2008; Mitri et al., 2011).

Kreft (2004) used IbM to study competition between the rate and yield strategists in biofilms and concluded that certain spatial structures are needed for maintenance of yield strategists. The rate strategists are found to dominate the biofilm in the short-term due to their high growth rates, while in the long run the yield strategists dominate since they consume nutrients more economically. Nadell et al. (2010) studied competition between enzyme secreting and non-secreting bacteria under different ratios between nutrient provision and nutrient consumption, and found that if the ratio is small, cell (bacteria) lineage segregation occurs and consequently the cooperative cells (i.e., enzyme-secreting cells) dominate within the biofilm. The cell lineage segregation confers an advantage to the cooperative cells because they are not exploited by non-cooperative ones. Mitri et al. (2011) found that addition of new species in a multispecies biofilm especially in resource limited scenarios would reduce the fitness of existing cooperative cells that secrete public goods. In addition, the ecological advantages of quorum sensing (QS) -regulated enzyme production (Schluter et al., 2016), QS inhibition (Wei et al., 2016) and evolution of bacteriocin production (Bucci et al., 2011) in biofilms have also been investigated using IbM.

EPS mediated adhesion is known to be very important for bacterial biofilm development as it affects both the initial attachment to surfaces and the subsequent resistance to shear flows. However, bacterial adhesion to surfaces ought to be costly because it restricts bacteria mobility and hinders movement to nutrient rich environments. Schluter et al. (2015) studied the effect of EPS mediated adhesion and found that cells with greater adhesive capabilities gained a competitive advantage when nutrients are abundant. Xavier and Foster (2007) showed that cells that constitutively produce EPS (EPS+) outcompete non-producers (EPS−) in the presence of significant nutrient gradients. When the EPS+ and EPS− strains are co-cultured in a biofilm, the EPS+ cells initially grow slower than EPS− cells because the EPS+ cells spend a fraction of energy on EPS production, and therefore the EPS− bacteria would initially dominate in the biofilm. However, eventually the production of EPS would help the EPS+ cells to push their descendants into nutrient rich top layers and hence the progeny of EPS+ bacteria would get more access to nutrients and would dominate in the biofilm in long run. Quorum sensing (QS) is a cell-cell communication mechanism used to regulate gene expression and production of public goods in biofilms (Fuqua and Greenberg, 2002). Nadell et al. (2008) investigated the competitive advantage of quorum sensing-mediated down regulation of EPS production. They found that EPS producers under negative quorum sensing control (i.e., EPS production by bacteria stops at high cell densities, referred to as the QS− strain), would dominate when competing with EPS+ strain. However, this effect only lasts for a limited time and the EPS+ cells dominate in the long-term because EPS+ cells suffocate the QS− cells by continuously secreting polymeric substance thereby separating QS− cells from nutrients.

These studies demonstrate that spatial distribution of microbes influences the microbial competition in biofilms. In addition nutrient gradients have been known to cause cell lineage segregation in biofilms and the effect has been addressed in many papers (Xavier and Foster, 2007; Nadell et al., 2010). Generally, low nutrient conditions favor cooperative strains (or species) that produce public goods such as EPS and enzymes. The biofilm structure is also influenced by other factors including microbial mobility, adhesion, initial attachment frequency and bacteria re-attachment to the biofilm (van Gestel et al., 2014); however, the effect of these factors on microbial competition in biofilms has not been extensively investigated. For example, when a biofilm grows in a reactor, it can experience erosion and sloughing due to hydrodynamic shearing and the detached biofilm clusters can re-colonize new surfaces and develop into biofilms. Similarly, the aerobic granular sludge aggregates found in sequencing batch reactors can be transported to new locations and have the ability to colonize new surfaces (McSwain et al., 2005). It is therefore very likely that bacterial aggregates deposit on new surfaces, hence biofilms originate from both individual cells (single cells) and cell clusters (aggregates). Only recently, Melaugh et al. (2016) and Kragh et al. (2016) addressed a similar problem by performing IbM simulations to understand the trade-off between aggregate surface area and relative height compared to single cell colonizers. The findings suggest that single cells perform better when competition is low (i.e., at low single cell densities) and multicellular rounded aggregates perform better when competition is high (i.e., at high single cell densities). In more competitive environments the aggregates perform better because they have access to nutrient rich areas due to their initial height advantage compared to single cells. This trade-off is likely to be influenced by EPS production characteristics of cells because EPS provides cells with sufficient structure to reach high nutrient layers. Moreover, multispecies biofilms may contain strains of bacteria that can either be EPS+ or EPS−. Therefore, EPS production characteristics of cells might offset the competitive advantage gained by bacterial aggregates due to their height.

In the present study, we develop a two-dimensional biofilm model based on IbM principles to understand competition between cells and aggregates which express a combination of characteristics (EPS+, EPS−, QS+ and QS−, described under “Methods” below). We simulate the spatiotemporal dynamics of competition under various scenarios of attachment (i.e., as single cells or multiple aggregates) and for different values of energy invested in EPS production by the microbes. The maximum competitive advantage is obtained when the EPS+ cells are initially deposited on the substratum as smaller aggregates and are randomly distributed among individual cells of the EPS−. We also study the effect of quorum sensing- regulated EPS production on competition between single cells and aggregates for different values of QS signal threshold. Overall, the work demonstrates the role of EPS production in conferring an advantage to either single cells or aggregates as they form biofilms under differing conditions.

## Methods

### Individual-based model

The components of the two-dimensional Ib model are similar to that used in Nadell et al. (2008). The bacteria are represented as hard spheres, each having variable mass/volume and a set of growth parameters. Each bacterium grows by consuming substrate (*S*) which is supplied from the bulk liquid. Four strains are considered: (i) EPS producer with no quorum sensing (EPS+), (ii) no EPS production, with no quorum sensing (EPS−), (iii) EPS producer under negative quorum sensing in which EPS secretion stops at high cell densities (QS−), and (iv) EPS producer under positive quorum sensing in which polymer secretion starts at high cell densities (QS+). The growth rate of a bacterium of any strain (EPS+, EPS−, QS+, QS−) having a mass of *m* is calculated as:

(1) $$\begin{array}{r} \frac{dm}{dt}=\left(\left(1-f\;Q\left(AI\right)\right)\mu_{\max}\frac{S}{K_{S}+S}-\frac{\sigma }{Y_{AX}}\right)m \end{array}$$

where μ_max_, *K*_*S*_, and *S* are the maximum specific growth rate, half saturation coefficient and local substrate concentration, respectively. σ and *Y*_*AX*_ are the production rate and corresponding yield of the quorum sensing signal (auto-inducer, *AI*). The EPS producing bacteria spend a fraction of the energy (*f*) gained from nutrients on EPS production and the remaining fraction (1−*f*) on growth and division. The value of the switching function *Q*(*AI*) is calculated as explained below. As shown in Table 1, all strains except EPS− can produce EPS and all strains produce *AI*. Over time EPS accumulates within the shells around the EPS producing cells and is subsequently excreted as EPS particles. Once a bacterium reaches a pre-determined cellular mass, it divides into two cells. The pressure build-up due to biomass growth is released by biofilm expansion (Kreft et al., 1998). The concentrations of substrate (*S*) and auto-inducer (*AI*) are calculated as:

(2) $$\begin{array}{r} \frac{\partial S}{\partial t}=D_{S}\nabla^{2}S-\frac{\mu_{\max}}{Y_{XS}}\frac{S}{K_{S}+S}X \end{array}$$

(3) $$\begin{array}{r} \frac{\partial AI}{\partial t}=D_{AI}\nabla^{2}AI+\sigma X \end{array}$$

where *X* is the local biomass concentration and *D* represents the diffusion coefficient of the respective solute. For the non-quorum sensing strains EPS+ and EPS− the function *Q*(*AI*) given in Equation (1) is independent of the *AI* signal and is always equal to 1 and 0, respectively. For the negative quorum sensing strain (QS−), *Q*(*AI*) = 1 if the quorum sensing signal concentration is less than the quorum sensing threshold τ and *Q*(*AI*) = 0 otherwise. For the positive quorum sensing strain (QS+), *Q*(*AI*) = 1 if the quorum sensing signal concentration is greater than the quorum sensing threshold τ and otherwise *Q*(*AI*) = 0.

**Table 1:** Stoichiometric table.

**Reaction**	**Soluble components**		**Particulate components**			**Rate expression**
	***S***	***AI***	**X_*EPS*+, *QS*+, *QS*−_**	**X_*EPS*−_**	**EPS**	
EPS+/QS+/QS− growth	$-\frac{1}{Y_{XS}}$		1 − *fQ*(*AI*)		*fQ*(*AI*)	$\mu_{\max}\frac{S}{K_{S}+S}X_{EPS+,QS+,QS-}$
EPS− growth	$-\frac{1}{Y_{XS}}$			1		$\mu_{\max}\frac{S}{K_{S}+S}X_{EPS-}$
*AI* production by EPS+/QS+/QS−		1	$-\frac{1}{Y_{AX}}$			σ*X*_*EPS*+, *QS*+, *QS*−_
*AI* production by EPS−		1		$-\frac{1}{Y_{AX}}$		σ*X*_*EPS*−_

*The substrate and auto inducer are considered as soluble components, while the bacteria and EPS are considered are particulate components. EPS+ cells invest a fraction (f) of energy on EPS production and the remaining 1-f goes for biomass production. Q = 1 for EPS+. For QS− strain, the function Q is equal to zero if auto inducer concentration AI is greater than a certain threshold value (τ) and otherwise Q is equal to 1 and it is other way around for QS+*.

The physical space in which the biofilm grows is represented by a rectangular space of 400 μm × 200 μm divided into a 200 × 100 computational grid. The *x* direction has periodic boundaries which means that a bacterium that is pushed beyond the boundary plane re-enters the domain through the opposite boundary plane. The *y* direction has the no-flux boundary condition at the substratum and Dirichlet boundary condition at the opposite end, which is the bulk liquid. Bacteria can spread toward the bulk liquid but not into the substratum (Kreft and Wimpenny, 2001). Equations (2) and (3) are solved for the steady state solution, as the rate of diffusion of solutes is very fast compared to the bacterial growth rate. Additional details about the model can be found in Xavier and Foster (2007) and Nadell et al. (2008).

### Numerical simulations and data analysis

At the beginning of any simulation the bacteria are placed on the inert, impermeable substratum located at *y* = 0 and are considered to be attached. Initially, 50 cells of each bacterial strain (EPS producing and non-producing) are placed on the substratum. The simulations are performed for a maximum of 12-days as the simulation box (400 μm × 200 μm) cannot accommodate larger biofilms. Similar to others (Xavier and Foster, 2007), the fitness of EPS+ is calculated as $w_{EPS+}=\log_{2}\left(\frac{N_{EPS+,t}}{N_{EPS+,t0}}\right)$, where *N*_*EPS*+, *t*0_ is the initial number of bacterial cells and *N*_*EPS*+, *t*_ represents the number of bacterial cells at a chosen time *t*. The fitness of EPS− is defined in the same manner and the relative fitness of EPS+ compared to EPS− is calculated as $w_{r}=\frac{w_{EPS+}}{w_{EPS-}}$. The fitness of QS+ and QS− strains relative to EPS− are defined in the same manner. The parameters used for the numerical simulations are listed in Table 2. Each simulation is replicated 10 times and the average is taken for the analysis. We also analyse the relationships between relative fitness of EPS+/QS+/QS− and various input variables discussed below by using generalized linear modeling (GLM) in the R statistical programming language.

**Table 2:** Parameters used for the simulations.

**Symbol**	**Description**	**Value**	**Reference**
τ	Quorum sensing threshold	5–10 × 10^−7^ kg/m^3^	Frederick et al., 2011
σ	AI production rate	1.7 × 10^−8^ s^−1^	Vaughan et al., 2010
*f*	Fraction of energy investment in EPS	0–0.6	Nadell et al., 2008
μ_max_	Maximum bacteria growth rate	1 h^−1^	Nadell et al., 2008
ρ	Biomass density	220 kg/m^3^	Kreft, 2004
ρ_*EPS*_	EPS density	33 kg/m^3^	Nadell et al., 2008
*D*_*S*_	Diffusivity of substrate	1.6 × 10^−9^ m^2^/s	Nadell et al., 2008
*D*_*AI*_	Diffusivity of AI	1.6 × 10^−9^ m^2^/s	Vaughan et al., 2010
*K*_*S*_	Half-saturation constant	3.5 × 10^−5^ kg/m^3^	Nadell et al., 2008
*Y*_*XS*_	Yield of biomass on substrate	0.5	Nadell et al., 2008
*Y*_*AX*_	Yield of auto-inducer on biomass	20	Nadell et al., 2008
*S*_*b*_	Bulk substrate concentration	5 × 10^−4^ kg/m^3^	Nadell et al., 2010
*L*	Boundary layer thickness	100 μm	–

## Results and discussion

In the following sections, the competition between various strains of bacteria (EPS+/QS+/QS−/EPS−) are investigated for a period of 12 days given that they initially attach on the surfaces as either cells or aggregates.

### Competition between EPS producing (EPS+) and EPS non-producing (EPS−) strains

**Figure 1:**
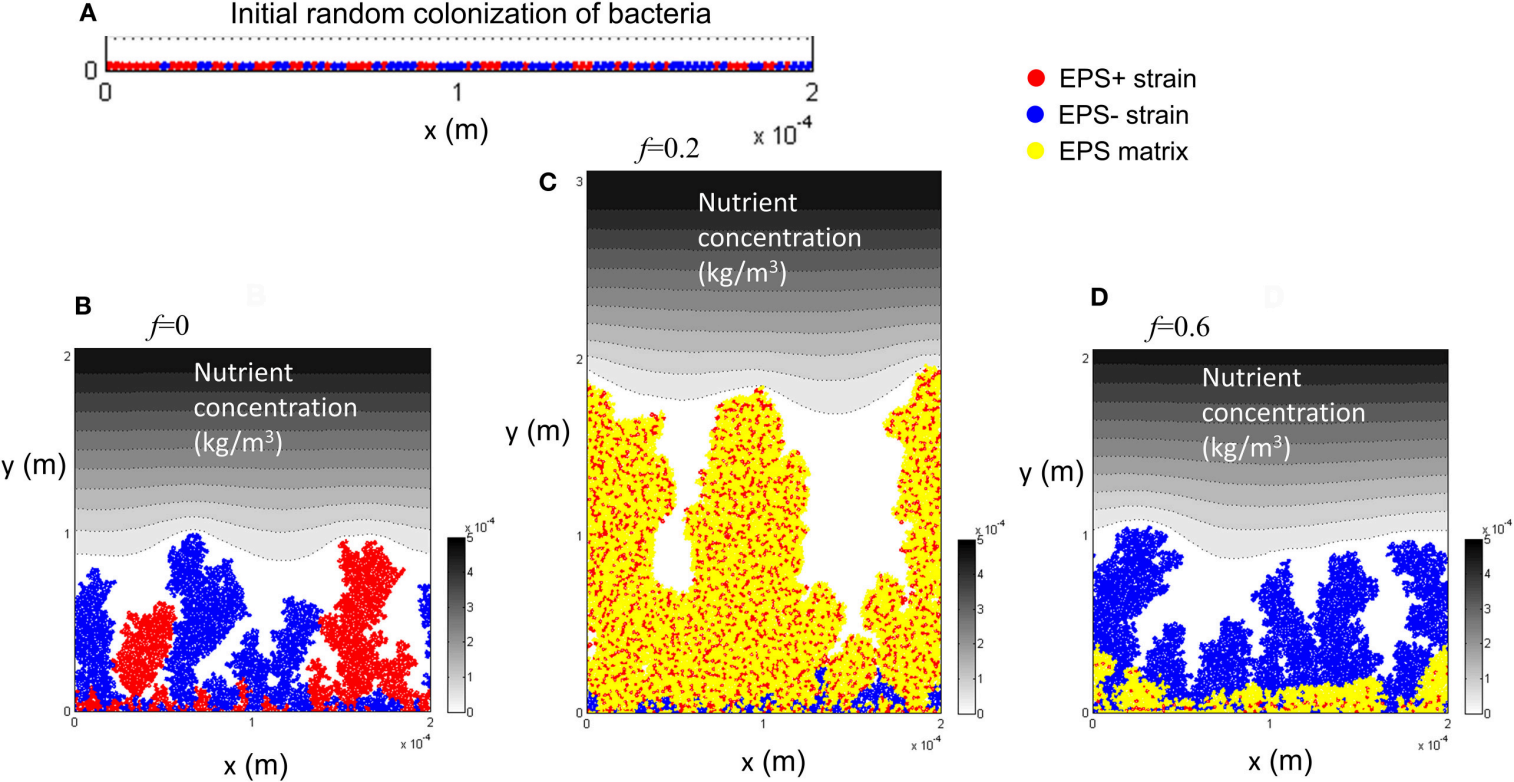
Competition between EPS+ and EPS− strains when both strains are initially randomly inoculated on the substratum: **(A)** initial inoculation of bacteria; **(B)** biofilm after 12 days at *f* = 0; **(C)** biofilm after 12 days at *f* = 0.2; **(D)** biofilm after 12 days at *f* = 0.6. It is seen that both strains co-exist in the biofilm if there is no EPS production, EPS+ strain dominates at *f* = 0.2 and EPS− strain dominates at *f* = 0.6. The contour plot shows the nutrient level from low to high as white to black. All values are in SI units.

**Figure 2:**
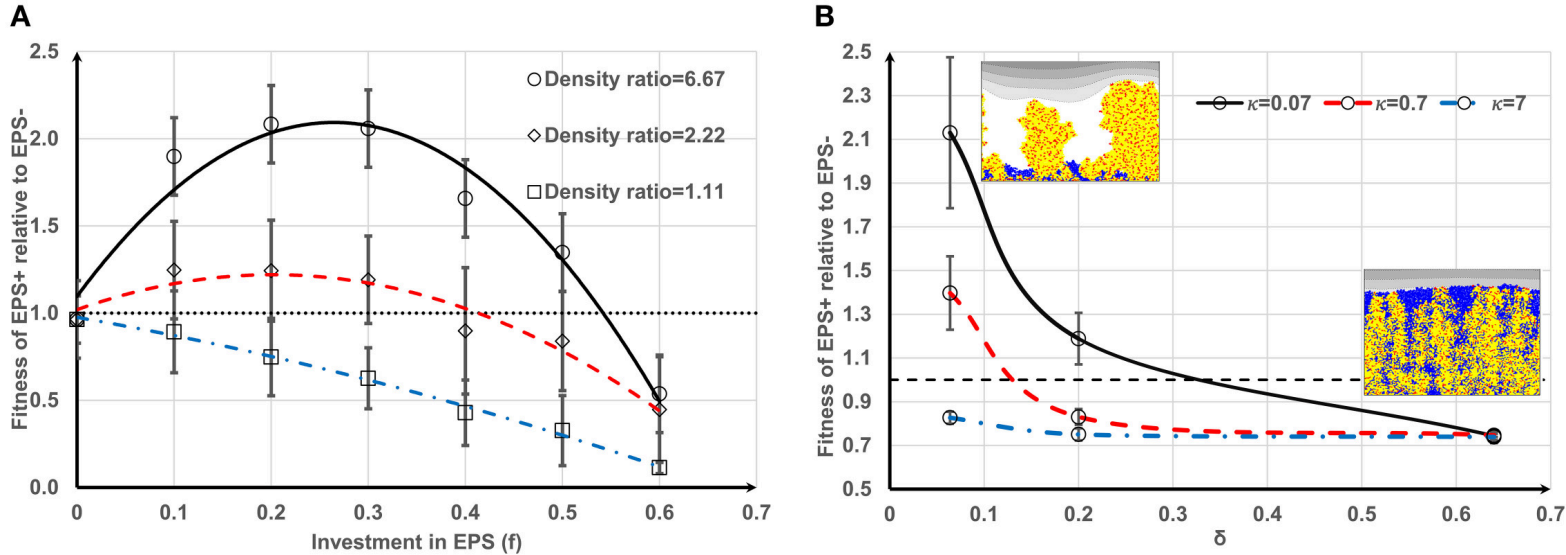
Effect of different parameters on the fitness of EPS+: **(A)** Fitness of EPS+ strain relative to EPS− strain as a function of investment in EPS (*f*) and biomass to EPS density ratio (ρ/ρ_*EPS*_). It is seen that if the density ratio is high it is advantageous for EPS+ strain and also there would be an optimum *f*-value which gives the maximum benefit for EPS+ strain. If the EPS density is relatively low, EPS+ cells are easily outcompeted by EPS− cells since EPS+ cells cannot push their progeny fast into the nutrient rich upper levels. The lines are the polynomial fits to the corresponding data points and the error bars indicate the standard deviations; **(B)** relative fitness of EPS+ strain relative to EPS− strain as a function of δ and κ which are two non-dimensional parameters appeared in the nutrient transport equation. It is clear that EPS+ are not beneficial at high values of δ and κ since the heterogeneity of nutrient concentration is less in this case and hence both strains are mixed in the biofilms rather than making own lineages. The lines are the polynomial fits to the corresponding data points and the error bars indicate the standard deviations.

Competition between EPS+ strain and EPS− strain when they initially deposit on the substratum as individual cells has been studied by Xavier and Foster (2007). A similar case is reproduced here as a control. The bacteria (50 EPS+ and 50 EPS−) are randomly inoculated on the substratum and all of the cells have equal access to substrate (at *t* = 0 s). Figure 1 shows the biofilm formation for different values of investment in EPS production (different *f*-values). It is seen that if there is no investment in EPS (*f* = 0, Figure 1B) both species grow identically and there is no competitive advantage for either. However, if energy investment in EPS production is relatively high (*f* = 0.6, Figure 1D), EPS+ strain is outcompeted by EPS− strain. At an intermediate fraction of energy investment (*f* = 0.2, Figure 1C), EPS+ cells dominate in the biofilm. The variation of relative fitness of EPS+ cells as a function of investment in EPS (*f*) and EPS material density ρ_*EPS*_ is shown in Figure 2A. If the density of EPS decreases compared to the density of bacteria (i.e., ratio ρ/ρ_*EPS*_ increases) it is advantageous for EPS+ cells since the volume of polymeric substances expands faster. This results in the EPS+ strain being pushed into substrate rich environments while EPS− cells are starved. Xavier and Foster (2007) also briefly demonstrated that the amount of substrate plays a vital role in the competition between EPS+ and EPS− strains. We find that the relationship between the ratio of the fitness of EPS producers to non-producers for different values of EPS investment (0 < *f* < 0.6) is unimodal for density ratio ρ/ρ_*EPS*_ > 2.2 (*t* = 6.745, *P* = 0 and *t* = −9.809, *P* = 0 respectively for the linear and quadratic terms for investment in EPS, *f*, more details about GLM are in Supporting information), indicating that above a certain threshold of investment in EPS the relative fitness of EPS producers declines. For low density ratio conditions (ρ/ρ_*EPS*_ < 2.2), the relative fitness of the EPS+ strain declines with increased investment in EPS.

To better understand the trade-off due to substrate limitation and bacteria growth, we direct our attention to the nutrient transport equation. (The density ratio for the following simulations is ρ/ρ_*EPS*_ = 6.67 which is estimated from the parameters in Table 2). For our model, the substrate gradients are determined by Equation (2), which can be re-written in non-dimensional form as:

(4) $$\begin{array}{r} \frac{\partial S^{*}}{\partial t^{*}}=\delta^{2}\nabla^{2}S^{*}-\frac{S^{*}}{\kappa +S^{*}}X^{*} \end{array}$$

where $S^{*}=S/S_{b}$ is the non-dimensional concentration and *S*_*b*_ denotes the bulk substrate concentration. $\delta =\sqrt{\frac{D_{S}Y_{XS}S_{b}}{\mu_{\max}\rho L^{2}}}$ and $\kappa =\frac{K_{S}}{S_{b}}$ are non-dimensional parameters, and ρ and *L* are biomass density and substrate concentration boundary layer thickness, respectively. The dimensionless parameter δ (Nadell et al., 2010) represents the ratio between the maximum rate of substrate transport and maximum rate at which substrate is consumed by bacteria. The biological meaning of κ is subtle: it expresses the affinity of the bacteria for a substrate in the context of given bulk substrate concentration.

It can be deduced from Equation (4) (if we only consider the *y* direction) that the steady state substrate transport is given by $\frac{d^{2}S^{*}}{dy^{*2}}=\frac{1}{\delta^{2}}\frac{S^{*}}{\kappa +S^{*}}X^{*}$, and thus the substrate gradient across the biofilm is $\frac{dS^{*}}{dy^{*}}=\frac{1}{\delta }\sqrt{2X^{*}(S^{*}-\kappa \ln(S^{*}+\kappa )+C}$, where *C* is a constant. It is obvious that the substrate gradients are negatively correlated with κ and δ. Increasing the value of either parameter would decrease substrate gradients and therefore result in substrate rich conditions throughout the biofilm. Figure 2B shows that when κ and δ increase, the EPS− strain easily outcompetes the EPS+ strain due to smaller substrate gradients across the biofilm. If κ is very high (κ = 7), the EPS− strain outcompetes the EPS+ strain regardless of δ. Increasing either parameter results in substrate rich conditions throughout the biofilm and results in a lack of lineage segregation in the biofilm. Since the EPS+ and EPS− strains are well mixed in the biofilm network the EPS+ cells can be exploited by EPS− cells. We were inspired by Nadell et al. (2013) to derive a simple relationship analogous to Hamilton's rule for the competition between EPS producers and non-producers to show that our model predictions (Figures 1, 2) are consistent with this rule. According to Hamilton's rule (Hamilton, 1964), a cooperative strategy, such as EPS production, will evolve if *rB* > *C*, where *r, B* and *C* are relatedness (measure of genetic similarity of the neighboring cells to the focal cell), fitness benefit, and fitness cost, respectively. The growth rate of a EPS+ cell can be written as $\frac{dm_{EPS+}}{dt}=\left[\mu_{0}\left(1+B\right)-f\right]m_{EPS+}$, where *B* is the additional benefit gained by the cell because the cell is advected to high nutrient layers by the polymeric substances and μ_0_ is the specific growth rate of the cell. A nearby EPS− cell will also be benefited by EPS production depending on how far that cell resides from the EPS+ cell. If we assume this EPS-mediated benefit is inversely proportional to the distance from the EPS+ cell (*d*), the growth rate of EPS− cell can be written as $\frac{dm_{EPS-}}{dt}=\left[\mu_{0}\left(1+Br_{EPS+}/d\right)\right]m_{EPS-}$, where *r*_*EPS*+_ is the radius of EPS+ cell. The EPS+ cell will outcompete EPS− cell if EPS+ cell has higher fitness and therefore:

(5) $$\begin{array}{r} \frac{1}{m_{EPS+}}\frac{dm_{EPS+}}{dt}>\frac{1}{m_{EPS-}}\frac{dm_{EPS-}}{dt} \end{array}$$

which gives that if $\left[\mu_{0}(1+B)-f]>[\mu_{0}(1+Br_{EPS+}/d\right]$. Therefore, the cooperative strategy will evolve if:

(6) $$\begin{array}{r} \left(1-r_{EPS+}/d\right)B>f/\mu_{0} \end{array}$$

The condition given in Equation (6) is analogous to Hamilton's rule, *rB* > *C*, with *r* = 1 − *r*_*EPS*+_/*d* and $C=f/\mu_{0}$. According to Equation (6), when *f* increases the relationship will fail at a point where the EPS− strain would outcompete the EPS+ strain. Figures 1, 2 clearly show this behavior. Equation (6) also indicates that EPS+ cells will dominate if EPS− cells are far away from the growing EPS+ cells (*d* >> *r*_*EPS*+_, meaning that relatedness is high). Figure 2B shows similar behavior, the EPS+ strain dominates when there is lineage segregation (for low κ and δ) and EPS− strain dominates when the two strains are mixed (for high κ and δ). Despite the simplicity of the current Ib model, it can predict the competition between polymer producers and non-producers in biofilms which is akin to Hamilton's rule.

### Competition between aggregates and cells (with EPS+/EPS− characteristics)

In reality, biofilms can be initiated by a mixture of single cells and aggregates. If there is a steep nutrient gradient across the biofilm (i.e., small κ and δ values), the initial colonization pattern (i.e., excess of aggregates or single cells) could have a profound effect on the fate of the biofilm inhabitants. Two recent studies (Kragh et al., 2016; Melaugh et al., 2016) that did not consider EPS production, found that bacteria attaching as aggregates would have a competitive advantage over single cells; as the height of the former gives better access to resources. This competition can be directly influenced by over expression of EPS in the aggregates which can provide them with even greater access to resources and thereby an even greater advantage. To investigate such scenarios we modeled the competition between EPS+ and EPS− bacteria when they attach on the substratum as either circular aggregates or individual cells.

We start the investigation by considering two different scenarios for the initial cell and aggregate attachment on the substratum:

(i) Case 1: EPS+ bacteria are deposited as aggregates and EPS− bacteria are distributed as single cells.

We consider the case in which EPS+ and EPS− cells deposit on the substratum as aggregates and single cells, respectively. The initial number of aggregates is varied between 1, 2, and 5 such that the cell number ratio between two strains is always 1:1. Therefore, as the number of aggregates increases, the size of each aggregate decreases accordingly (Figure S1). Given the pattern of initial colonization, EPS+ aggregates should have two distinct advantages: as the aggregates produce EPS they can suffocate EPS−, and they can use their height advantage to obtain improved access to substrate.

**Figure 3:**
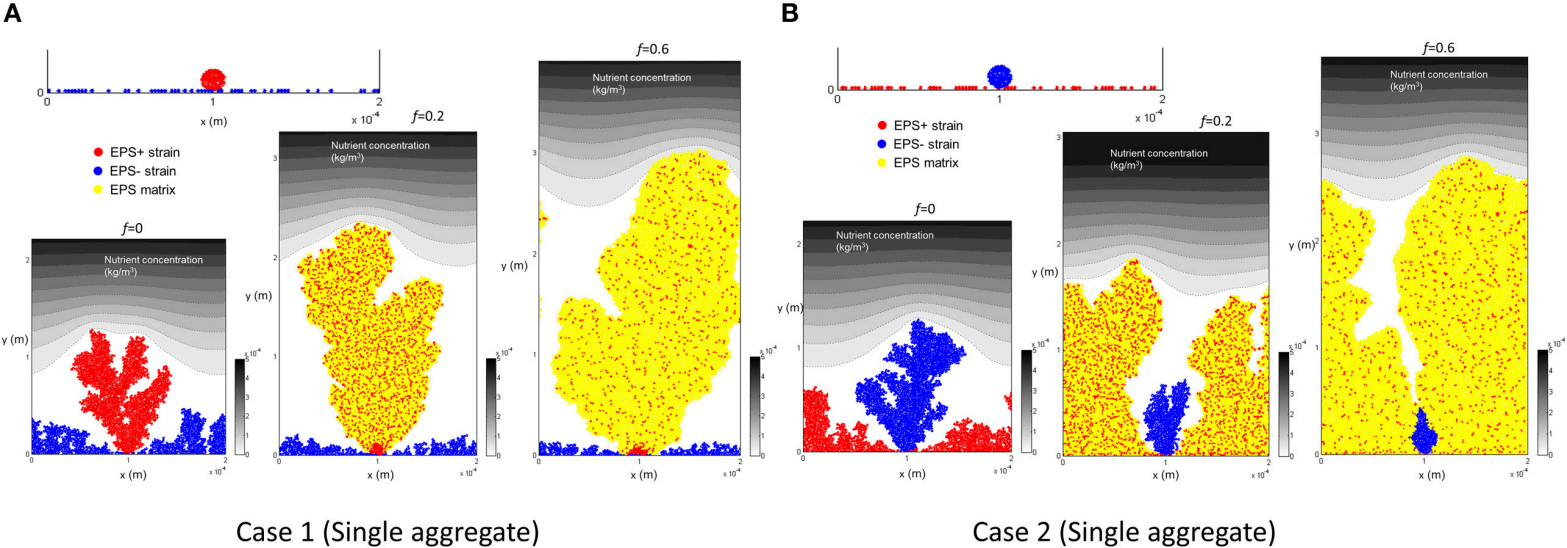
Initial colonization of one aggregate and biofilm after 12 days for different level of energy investment in EPS (*f*): **(A)** Case 1, EPS+ cells are initially aggregated while EPS− cells are randomly spread out on the substratum. It is seen that EPS+ strain dominates in the biofilm for all the cases; **(B)** Case 2, EPS+ cells are randomly spread out on the substratum while EPS− cells are initially aggregated. It is seen that EPS+ strain dominates in the biofilm for all the cases. All values are in SI units.

Figure 3A shows biofilm growth when EPS+ strain deposits as a single aggregate. We find that EPS+ strain grows as a single tower and the growth of EPS− cells is inhibited. The population density of EPS+ cells in the EPS matrix decreases as *f* (the fraction of energy devoted to EPS production) increases.

(ii) Case 2: EPS+ bacteria are spread out as single cells and EPS− bacteria are deposited as aggregates.

We consider the situation in which the EPS− and EPS+ cells deposit on the substratum as aggregates and single cells, respectively. Similar to Case 1, the number of aggregates is varied as 1, 2, and 5 while maintaining the 1:1 ratio between the strains. Even though EPS− cells do not produce EPS, they are still likely to aggregate due to pili-pili interactions between bacterial cells (Ponisch et al., 2017). Aggregates of EPS− may have a competitive advantage over EPS+ cells due their height and better access to nutrients, however the EPS+ cells may gain a competitive advantage by producing EPS.

Figure 3B shows the results when EPS− strain deposits as a single aggregate. We find that, although EPS− cells are initially aggregated and have some competitive advantage due to height, EPS+ cells always dominate in the biofilm. As the energy invested in EPS production is relatively high (*f* = 0.6), the EPS− tower is surrounded by the polymeric matrix due to rapid EPS production and hence EPS− aggregate is not able to access nutrients.

**Figure 4:**
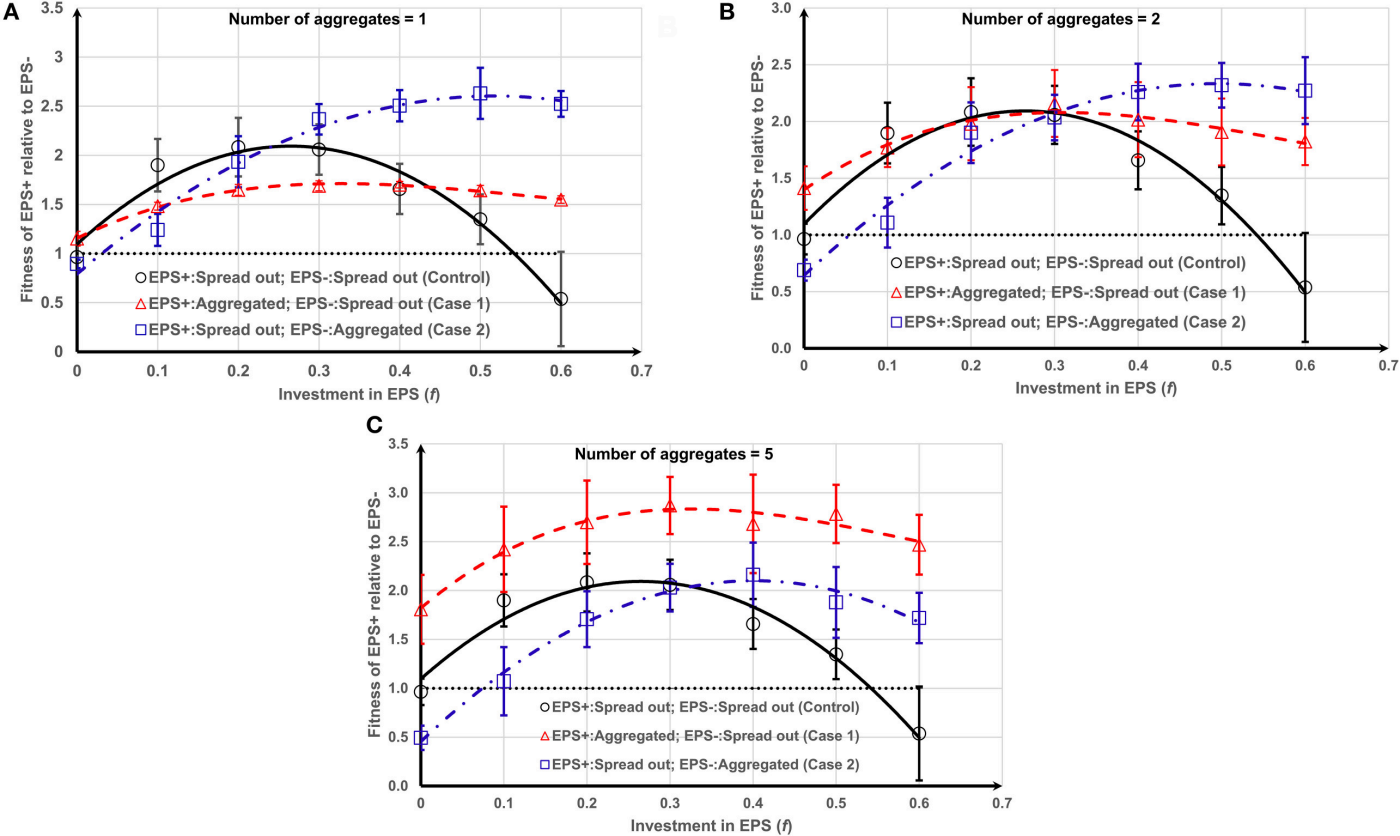
Fitness of EPS+ relative to EPS− as a function of *f* and initial inoculation and number (or size) of aggregates (see Figure S2 for different cases): **(A)** one aggregate; **(B)** two aggregates; **(C)** five aggregates. EPS+ strains get the maximum benefit if EPS− and EPS+ strains are initially spread out and aggregated, respectively. The lines are the polynomial fits to the corresponding data points and the error bars indicate the standard deviations.

The variation in the relative fitness of EPS+ cells for both cases (i and ii) is shown in Figure 4. At relatively low values of EPS investment (*f* < 0.25), starting as a single aggregate (EPS+ or EPS−) decreases the relative fitness of EPS+ bacteria when compared to both strains starting as single cells (Figure 4A). However, with greater EPS investment (*f* > 0.45), the relative fitness of EPS+ strain is significantly enhanced even though EPS− cells gain a height advantage by starting out as an aggregate. An increase in the number of aggregates results in the relative fitness curves moving upward and downward for Case 1 or Case 2, respectively (Figure S2), indicating that the number of aggregates have a significant effect on the competition between these two strains. As the number of aggregates increases (i.e., size of each aggregate decreases), the initially aggregated strain receives competitive advantage over the other strain.

EPS production (*f* > 0), no matter how modest, is better than no EPS production in nearly all situations as it allows better access to nutrients, suggesting that, if bacteria can produce EPS, they should. Our results show that the EPS+ strain obtains the maximum competitive advantage (Figures 4A,B) at *f* = 0.5 ± 0.1 (*P* = 0.0131) when EPS− strain is initially deposited as one/two aggregates and EPS+ strain is deposited as single cells. However, as the number of aggregates increases to five (Figure 4C) the EPS+ strain obtains the maximum competitive advantage at around *f* = 0.3 ± 0.2 (*P* = 0.0146) when EPS+ cells are initially deposited as aggregates and EPS− strain as single cells.

Generalized linear modeling for the data shown in Figure 4 was also performed to test the statistical significant of the results as detailed in the Supporting information. Case 1 with higher numbers of aggregates have higher relative fitness for EPS+ strain than either control or Case 2 (*t* = 9.737, *P* < 2 × 10^−16^). This indicates that EPS+ aggregates that are spread out more widely across the substratum relative to the non-EPS producers have a fitness advantage compared to when they are clumped into one colony, relative to non-EPS producers or when they are both distributed as single cells on the surface. The optimum EPS investment to maximize the relative fitness of EPS producers is clearly dependent on the spread and size of aggregates in the initial population.

**Figure 5:**
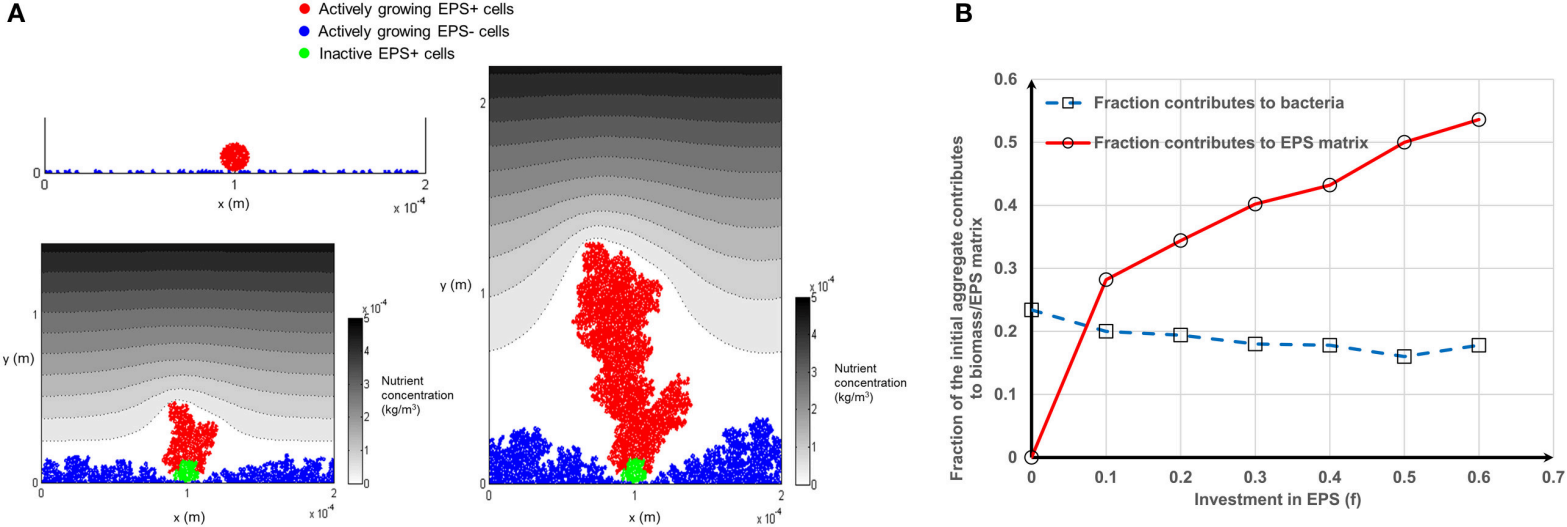
Inactive bacteria in the aggregate for Case 1: **(A)** As the biofilm grow some inactive bacteria are seen at the bottom of the aggregate. The inactive bacteria of the aggregate are shown in green color and these inactive bacteria would not contribute to biomass production (*f* = 0). Even though the aggregate gets a competitive advantage through its height the inactive cells in the bottom of the aggregate would be costly for it: **(B)** fraction of the active bacteria in the initially aggregated EPS+ strain as a function of investment in EPS production. As the investment in EPS increases from 0 to 0.6 the fraction contributes to bacteria production remains fairly constant around 0.2 and the fraction contributes to EPS production increases to around 0.55. Mass of the bacteria is the sum of the mass of EPS+ and EPS− strains. All values are in SI units.

The variation in fitness curves seen for Case 1 and Case 2 (Figure 4) for different numbers of aggregates can be further explained by scrutinizing the contribution of the initial aggregate to the biomass (mass of EPS+/EPS− cells) and EPS production over time. Bacteria at the bottom of the aggregate do not contribute to biomass production, irrespective of their status (EPS+ or EPS−) because they do not get sufficient substrate (Figure 5A). This limits the ability of a tall aggregate to compete with the singleton cells that surround it. When EPS investment increases from *f* = 0–0.6, the fraction of aggregate which contributes to EPS production increases from zero to around 0.55; while the fraction of aggregate which contributes to bacteria production hovers around a value of 0.2 (Figure 5B). Since the production of a unit volume of EPS is less expensive than the production of biomass (EPS+ strain) (material density of EPS is smaller than that of biomass, Table 2); it is easier for cells to directly invest in EPS production rather than creating new EPS+ cells. Separately, Figure 4 shows that the initially aggregated strain can obtain a fitness advantage by a greater margin if that strain deposits as smaller aggregates (Figures 4B,C). When the aggregate size decreases, the inactive bacteria seen in the initial aggregate (Figure 5) also decreases and hence a greater number of cells of the aggregated strain are available to actively compete with the other strain.

For Case 1, when EPS+ and EPS− strains are deposited as aggregates and single cells respectively, the EPS− cells are outcompeted by EPS+ cells over the whole range of *f* values (0 < *f* < 0.6). However, in Case 2, the distributed EPS+ cells can be outcompeted by EPS− aggregates if they do not produce enough EPS (*f* < 0.1, Figure 4C). This is in contrast to the control case (single cell attachment) where the EPS− cells can “catch a ride” on the polymeric material only when EPS+ cells heavily invest on EPS (when *f* > 0.5) and get lifted toward the nutrient rich surface (see Figures 1, 4), thereby gaining an advantage over the EPS+ cells. The segregation of the EPS+ and EPS− strains, as observed in Case 1 and Case 2, prevents the non-producers from being pushed to the top by the EPS+ neighbors investing heavily (*f* > 0.5) in production of polymeric material.

**Figure 6:**
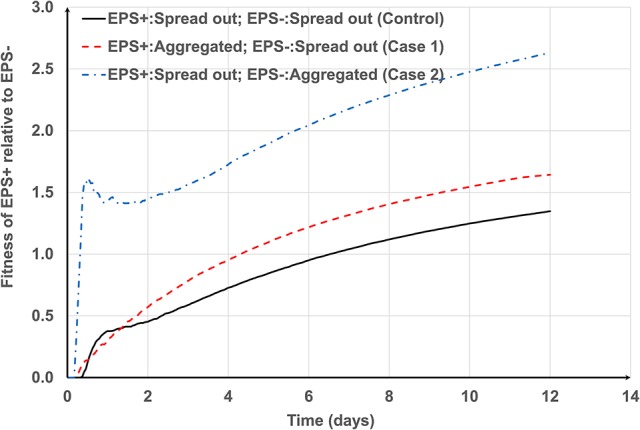
Transient variation of the fitness of EPS+ relative to EPS− at *f* = 0.5. The general trends for control case and Case 1 are similar. However, the fitness of EPS+ rapidly increases to 1.5 for Case 2 and then temporarily decreases and again increases at the same rate as other cases.

For smaller aggregates (Figure 4C) the greatest relative fitness for EPS+ is observed when EPS+ cells (around *f* = 0.3) are initially deposited as aggregates and EPS− strains are deposited as single cells (Case 1). This is expected because the EPS+ strain gains competitive advantage owing to its moderate height (even though they have a fraction of inactive bacteria) and ability to produce EPS. However, for larger aggregates (Figures 4A,B), the greatest relative advantage is observed when the EPS producers (around *f* = 0.5) are single cells and the non-producers are aggregated (Case 2) and this seems counter-intuitive. On closer inspection of time dependent relative fitness of EPS+ (*f* = 0.5, Case2, one aggregate) we find that it increases rapidly to around 1.5 at time < 1day, and then decreases transiently, before increasing again at the same rate as the other two scenarios (Figure 6). Therefore, the overall superior fitness of the EPS+ strain at *f* = 0.5 (Figures 4A,B) at day 12 can be attributed to the initial boost in fitness for the cells as seen in Figure 6. The reason for this initial fitness boost for EPS+ cells is two-fold: larger EPS− aggregates can have many inactive cells, and all EPS+ cells initially have good access to nutrients, hence they grow well. The initial fitness boost increases as *f* decreases since the EPS+ strain can invest more energy on production of EPS+ cells than polymeric substance. However, at low *f*-values, the EPS+ strain cannot maintain this initial boost for long since the EPS+ colonies cannot expand quickly due to lack of EPS.

Overall, the microbes with EPS+ characteristics gain a better competitive advantage if they initially colonize the surface as smaller aggregates and are widely spaced between the cells of EPS−. As the aggregate size decreases the EPS producing strain dominates in the biofilm even with lower levels of EPS production.

### Competition between QS+/QS− and EPS− strains

While EPS production is advantageous it is also metabolically expensive, and therefore it should be beneficial for its production in bacteria to be regulated through a feedback control mechanism such as quorum sensing. Two quorum sensing settings are considered in this work. In the first setting, QS− cells compete with EPS− cells and in the second setting QS+ cells compete with EPS− cells. For the sake of simplicity, the study is carried out at *f* = 0.5 which gives better fitness for EPS producers for a single aggregate deposition (Figure 4A). We examine the effects of different QS threshold values on the relative fitness of QS− and QS+ strains for the three scenarios mentioned above (control, Case1 and Case 2) but focus only on the case of a single aggregate (Figure S1).

**Figure 7:**
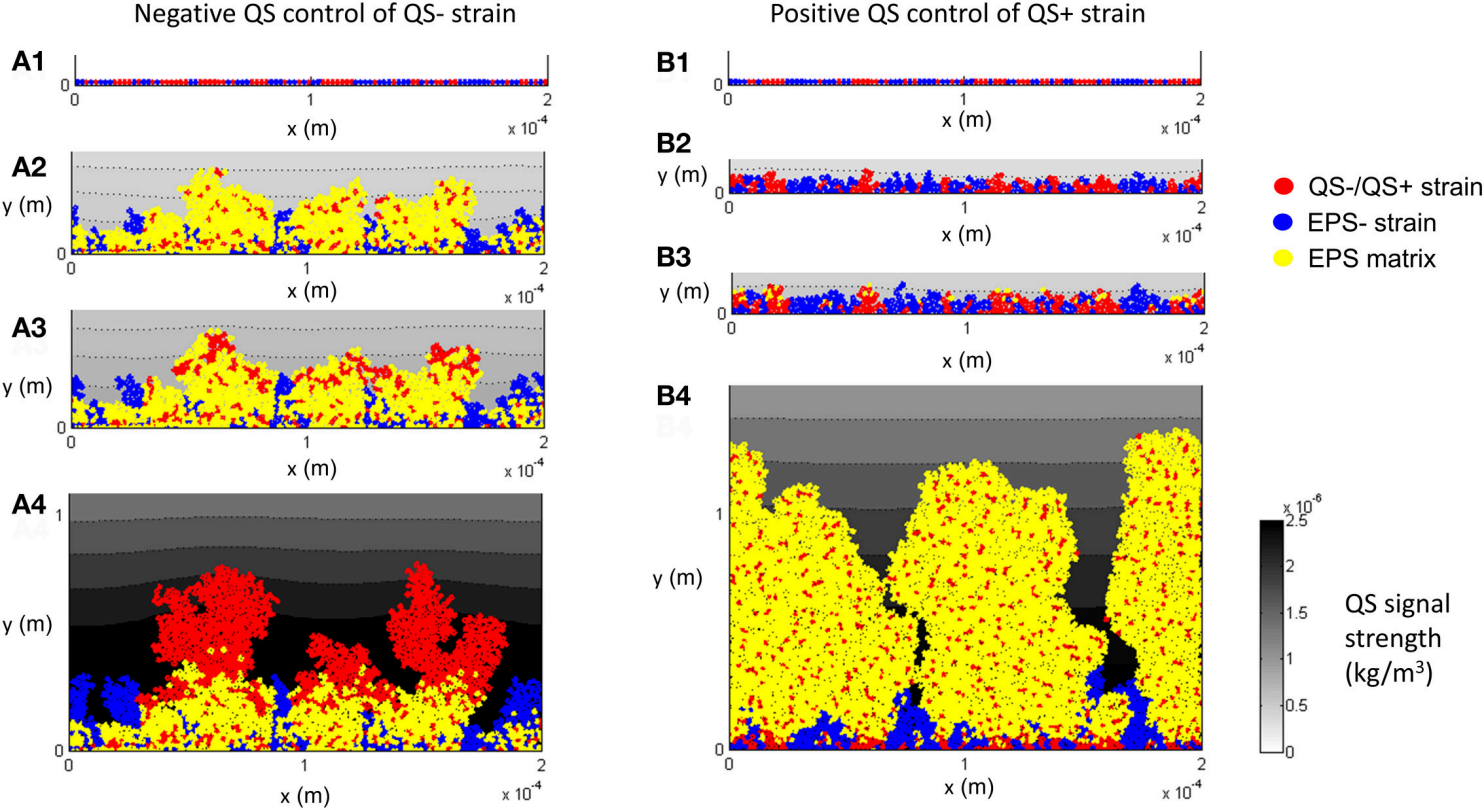
EPS production is negatively **(A1–A4)** and positively **(B1–B4)** controlled through quorum sensing (*f* = 0.5, τ = 5 × 10^−7^ kg/m^3^): **(A1)** both QS− and EPS− strains are randomly spread out on the substratum as individual cells; **(A2)** biofilm after 1.7 days; **(A3)** biofilm after 2.3 days, the QS− cells on the top of the biofilm gradually terminate the production of EPS; **(A4)** biofilm after 7 days, there is no EPS on the top of QS− linages. **(B1)** both QS+ and EPS− are randomly spread out on the substratum as individual cells; **(B2)** biofilm after 1.0 days; **(B3)** biofilm after 1.3 days, QS+ strain starts to produce EPS; **(B4)** biofilm after 7 days, QS+ strain dominates in the biofilm due to EPS matrix. All values are in SI units.

Figure 7 shows the diffusion of AI from the biofilm to the surrounding liquid and the resulting QS−regulation for the control case. Starting with single cells being deposited on the substratum (Figure 7A1), the population of QS− increases (Figure 7A2) and reaches the threshold for AI, τ = 5 × 10^−7^ kg/m^3^ (Figure 7A3), then EPS production is terminated but the QS− cells (colored red in Figure 7A4) continue to proliferate under the negative QS control. For the positive QS control, starting from single cells (Figure 7B1), initial growth (duration < 1.3 days) of both strains is similar because their characteristics are identical when there is no EPS production (Figure 7B2). The QS+ strain then starts to produce EPS when AI reaches its threshold of τ = 5 × 10^−7^ kg/m^3^ (Figure 7B3) and subsequently the QS+ strain dominates in the biofilm because they gain a competitive advantage due to formation of EPS matrix (Figure 7B4).

We compare three cell deposition scenarios (control, Case 1 and Case 2) when EPS production is regulated through QS, and examined the effect of threshold concentration of the auto-inducer (τ = 1 × 10^−7^, 5 × 10^−7^, 8 × 10^−7^, and 10 × 10^−7^ kg/m^3^) on the fitness of different strains.

**Figure 8:**
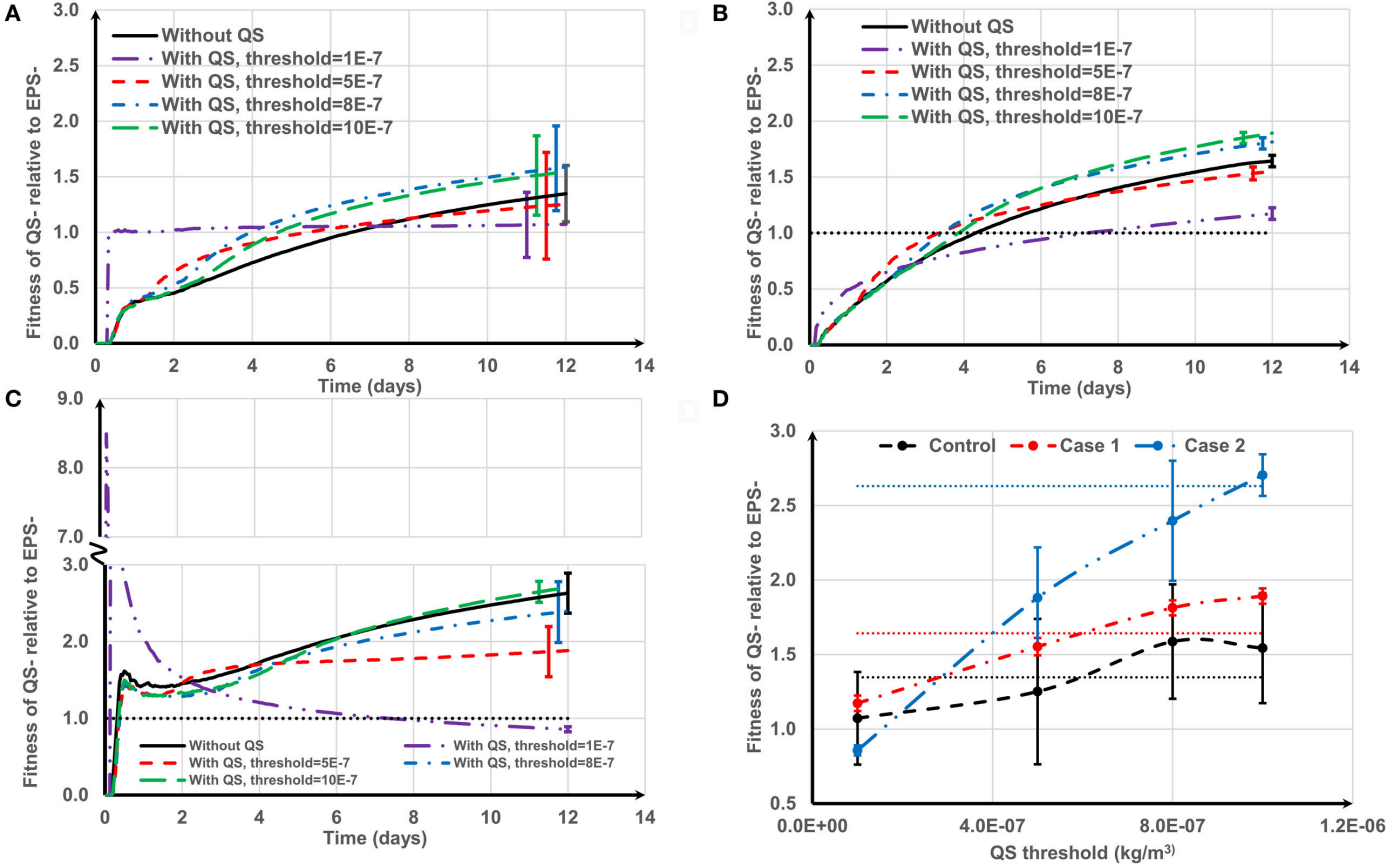
The effect of negative quorum sensing regulation on relative fitness of QS− strain at *f* = 0.5: **(A)** control case, both QS− and EPS− strains are initially spread out on the substratum; **(B)** Case 1, QS− cells are initially aggregated while EPS− cells are spread out; **(C)** Case 2, QS− cells are initially spread out while EPS− strains are aggregated. At the lowest threshold, the relative fitness rapidly increases to 8.5 and then decreases; **(D)** relative fitness of QS− strain on day 12. The dotted lines in **(D)** are the relative fitness without QS for respective cases. The standard deviations of plots **(A–C)** are shown only at the end for clarity. The standard deviation increases over time.

Figure 8 shows how the negative QS control affects the relative fitness of QS− strains for three cell deposition scenarios. The relationship between the fitness of EPS producers relative to non-producers under different initial deposition scenarios is significantly related to the threshold value of the auto-inducer concentration (*t* = 12.141, *P* = 0).

For the control case (Figure 8A), at the lowest threshold (τ = 1 × 10^−7^ kg/m^3^) the relative fitness is around 1 over time. This is because the QS− strain quickly reaches the QS threshold and terminates EPS production. Both QS− and EPS− strains become biologically identical and hence the relative fitness is around 1. At moderate thresholds (τ = 5 × 10^−7^ kg/m^3^) the fitness of QS− is only enhanced in the early stages of biofilm growth. However, higher thresholds (τ = 8 × 10^−7^ and 10 × 10^−7^ kg/m^3^) consistently improve QS− strain fitness. Low thresholds confer an initial short-term advantage followed by consistent reduction in fitness; higher thresholds confer a long-term advantage which is consistent with the findings of Nadell et al. (2008). The QS influence for Case 1 (QS− aggregate vs. EPS− single cells, Figure 8B) is analogous to the control case, except at the lowest threshold. At the lowest threshold the QS− strain stops production at the onset of growth; they only have the height advantage (Case 1) and therefore take longer to dominate the biofilm.

The benefit of QS in Case 2 (EPS− aggregate vs. QS− single cells, Figure 8C) is either negative or marginally positive. Quorum sensing is only of long-term value at the highest threshold (τ = 10 × 10^−7^ kg/m^3^). At the lowest threshold (τ = 1 × 10^−7^ kg/m^3^) the relative fitness of QS− strain rapidly increases to 8.5 and then gradually decreases and finally becomes negative. QS− cells stop producing polymeric substance in the beginning, and then the growth of the QS− strain is boosted (τ = 1 × 10^−7^ kg/m^3^). The reason behind this initial fitness boost for the QS− strain has already been explained in Figure 6. At the lowest threshold, EPS− cells dominate in the long run because they have a competitive advantage due to their initial height.

Figure 8D indicates that the long-term fitness of the QS− strain is more sensitive to the QS threshold for Case 2, but less sensitive for the other two cases. Moreover, for all the cell deposition scenarios, the relative fitness of the QS− strain is positively correlated with the QS threshold (the Pearson's correlation coefficients are 0.9512, 0.9921, and 0.9927 for control, Case 1 and Case 2, respectively). QS− cells are not outcompeted for the whole range of QS thresholds for the control and Case 1. Since QS− cells terminate EPS production at the onset of growth at the lowest threshold, both strains are identical for the control case and QS− cells have the height advantage for Case 1 (see Figure 4, control and Case 1 at *f* = 0). However, in Case 2, when QS− cells stop EPS production at the onset of growth, the QS− strain is easily outcompeted by EPS− due to the height advantage of the EPS− (see Figure 4, Case 2 at *f* = 0). At the highest QS threshold (τ = 10 × 10^−7^ kg/m^3^), although the relative fitness of QS− strain is at least slightly enhanced compared to the strains without QS for all attachment scenarios, only for Case 1 can we guarantee that QS− benefits from quorum sensing (*P* = 2.38E-9). This is because at higher thresholds, the QS− strain terminates EPS production after they dominate the biofilm, hence stopping EPS production may give a definite advantage to QS− strain for Case 1 because this strain also has the height advantage due to its initial aggregate nature.

**Figure 9:**
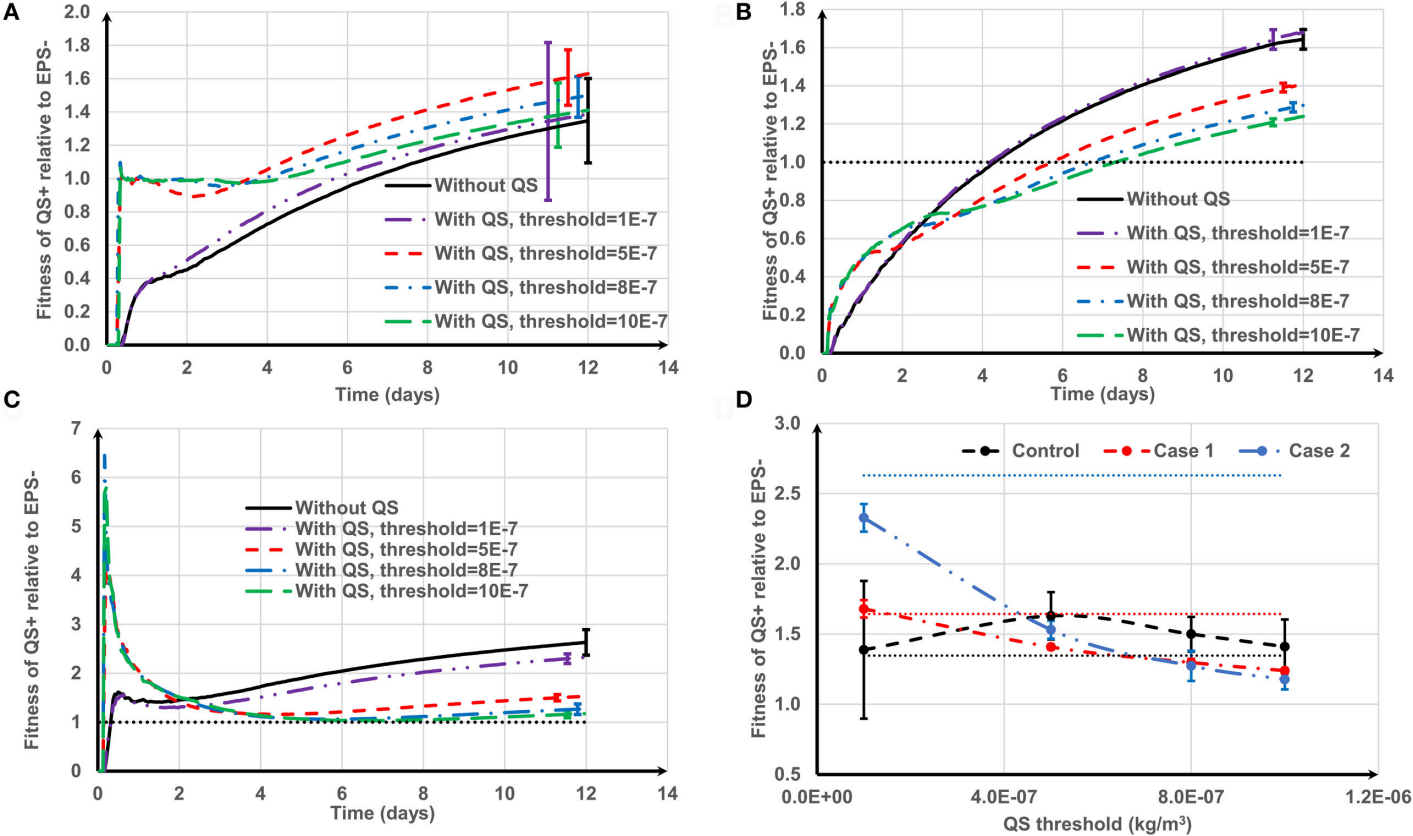
The effect of positive quorum sensing regulation on relative fitness of QS+ strain at *f* = 0.5: **(A)** control case, both QS+ and EPS− strains are initially spread out on the substratum; **(B)** Case 1, QS+ cells are initially aggregated while EPS− strains are spread out; **(C)** Case 2, QS+ cells are initially spread out while EPS− strains are aggregated. At the highest threshold, the relative fitness rapidly increases to 6 and then decreases; **(D)** relative fitness of QS+ strain on day 12. The dotted lines in **(D)** are the relative fitness without QS for respective cases. The standard deviations of plots **(A–C)** are shown only at the end for clarity. The standard deviation increases over time.

Figure 9 shows how the positive QS control affects the relative fitness of EPS producing strains for the three cell deposition scenarios. For the control (Figure 9A), the quorum sensing- regulated EPS production marginally enhances the relative fitness of QS+ strain for the whole range of QS thresholds (10^−7^ < τ < 10^−6^ kg/m^3^). At higher thresholds (τ >1 × 10^−7^ kg/m^3^), the QS+ strain does not produce EPS for a long time and thus the relative fitness of QS+ strain is around 1 until they start to produce EPS, and subsequently their fitness increases once EPS production commences. However, at the lowest threshold (τ = 1 × 10^−7^ kg/m^3^), EPS production starts quickly and so the QS+ strain needs time (about 6 days, Figure 9A) to dominate in the biofilm because it invests energy on both EPS matrix and QS+ cells, which is analogous to biofilm growth without quorum sensing regulation. There is an optimum QS threshold value for the control case at around τ = 5 × 10^−7^ kg/m^3^ (*P* = 0.006) (Figure 9D). However, for Case 1 and Case 2 (either strain initially deposited as an aggregate), the positive QS regulation of EPS is advantageous only at the beginning of biofilm growth (Figures 9B,C). The long-term relative fitness of the QS+ strain decreases as the QS threshold increases (*P* < 0.005) (Figure 9D). Therefore, the relative fitness of the QS+ strain is negatively correlated with QS threshold for Case 1 and Case 2 (the respective Pearson's correlation coefficients are −0.9867 and −0.9644).

Generalized linear modeling was used to investigate the collective effects of aggregate type, quorum sensing threshold and the occurrence of positive or negative regulation on the relative fitness of EPS producers compared to non-producers as detailed in the Supporting information. The detrimental effect of the quorum sensing threshold (*t* = −6.540, *P* = 3.83 × 10^−10^) and the occurrence of positive vs. negative control of EPS production (*t* = −10.248, *P* < 2 × 10^−16^) on relative fitness is very significant. There is a significant correlation between the quorum sensing threshold and whether the EPS is positively or negatively regulated (*t* = 13.868, *P* < 2 × 10^−16^) indicating a synergy between the two variables in their effects on fitness. The relative fitness of EPS producers is also dependent on the nature of cell deposition, with aggregated EPS producers (Case 2) resulting in higher fitness than the other two deposition scenarios (*t* = 6.660, *P* = 1.94 × 10^−10^).

Overall, we conclude that quorum sensing-regulated EPS production would enhance the fitness of EPS producers only marginally, or even reduces their competitive advantage, under the investigated conditions. This analysis shows that quorum sensing-mediated gene regulation in bacteria may be detrimental at times depending on the nature of the competition. Zhao and Wang (2017) argued that depending on the conditions there would be a “*right time*” and “*right place*” in which QS−regulated EPS production can favor biofilm growth; otherwise it would have unfavorable consequences for the EPS producers. Numerical experiments of Frederick et al. (2011) also show that QS−regulated EPS production rarely facilitates a biofilm to achieve a high cell population. However, maximizing offspring generation is not the only strategy bacteria may have, and sometimes production of EPS is beneficial if the objective is to produce a thick EPS protective layer. Therefore, further studies are needed to understand the role of QS− regulated EPS production for the cell deposition scenarios investigated here, taking into account the multiple functional roles of EPS in bacterial biofilms.

## Conclusions

Microbial competition between two bacterial strains with differing EPS producing characteristics (EPS+/QS+/QS− vs. EPS−), has been studied using an IbM, with one strain initially deposited on the substratum as aggregate(s) and the other as individual cells. The results show that when there is no quorum sensing and if EPS− cells attach as relatively large aggregates; then the EPS+ cells gain the maximum competitive advantage if they attach on the substratum as single cells (under the condition that the EPS+ strain invests about half of their energy in EPS production). Xavier and Foster (2007) and Nadell et al. (2008) also showed that the optimum investment in EPS is around 0.5, when EPS+ compete with others that invest either more or less in EPS production (in these studies, both strains are initially deposited as single cells on the surface, similar to the control case in this paper). However, when the EPS+ strain is deposited in relatively small clusters and the EPS− strain is deposited as single cells, then the EPS+ bacteria always benefit from producing EPS regardless of the level of energy invested in EPS. According to this simulation, as the EPS+ aggregate size decreases they need to expend less energy on EPS production (*f* < 0.5) to gain the maximum fitness advantage.

Quorum sensing-regulated EPS production is found to provide no significant advantage over continuous EPS production for all of the cell deposition scenarios, for the range of parameters chosen for the present study. Our numerical results indicate that quorum sensing-regulated EPS production significantly reduces the competitive advantage gained by matrix producers when they deposit as aggregates and compete with single cells of EPS− or vice-versa.

Laser-diffraction particle-size scanning tests have shown that 90% of the total planktonic biomass of *Pseudomonas aeruginosa* consist of cellular aggregates in the size range of 10–400 μm (Schleheck et al., 2009). Therefore, it is inevitable that single cells deposited on a surface will compete with different sizes of bacterial aggregates of *P. aeruginosa* which are deposited on the same surface. Our simulation results may give an insight into this competition because the present results indicate that the aggregate size plays a significant role in the competition with single cells. *In vitro* experiments of Kragh et al. (2016) have shown that aggregates of *P. aeruginosa* gain a competitive advantage over their single cells when competing in the same environment. These experiments could be extended to investigate the effects of different EPS production characteristics and different aggregate sizes on microbial competitions in biofilms, and then our predictions could be tested.

Wessel et al. (2014) used a gelatin based three-dimensional printing strategy to make different sizes of *P. aeruginosa* aggregate and showed that when the aggregate size exceeds a critical size, localized oxygen depletion regions were found inside the aggregate. These *in vitro* experimental results show that the growth rate decreases as the aggregate size increases which is consistent with our findings. Although the experimental and simulation results based on continuous model have general agreements, there was some discrepancy due to simplifying assumptions including uniform oxygen consumption throughout the aggregate. However, an Individual-based modeling technique similar to the present study should give more comparable results to these experiments because the IbM can capture heterogeneities inside aggregates more accurately. The present simulation techniques can also be adapted to study the interaction of bacteria such as *Sinorhizobium meliloti* that forms aggregates (Dorken et al., 2012) with other species during the wastewater treatment process (Ben Rebah et al., 2002).

Even though it is widely believed that public goods producing bacteria are benefited by quorum sensing-regulated gene modulations, our numerical results show that quorum sensing can also have detrimental effects on public good producers. However, these numerical simulations need to be extended to cover a wider range of parameters and be experimentally tested to draw a solid conclusion about these findings.

In the present Individual-based Model, factors such as bacteria motility, founder cell density, detachment and attachment etc. are not considered and these developments can form the basis for future work. Moreover, for biofilms growing in a flow environment, the mechanical strength of the biofilm mediated by the EPS composition can provide insights into the biological evolution of polymer producing strains. The flow can also advect quorum sensing signals which can cause the bacteria to misread their local cell density, thereby influencing bacterial competition in constricted geometries, for example in the pores of the soil.

## Author contributions

All authors listed, have made substantial, direct and intellectual contribution to the work, and approved it for publication.

### Conflict of interest statement

The authors declare that the research was conducted in the absence of any commercial or financial relationships that could be construed as a potential conflict of interest.

## Supplementary material

Data supporting this publication is openly available under an ‘Open Data Commons Open Database License’. Additional metadata are available at: https://doi.org/10.17634/123172-2. Please contact Newcastle Research Data Service at rdm@ncl.ac.uk for access instructions.
