# Supplementary material for: Extracellular Polymeric Substance Production and Aggregated Bacteria Colonization Influence the Competition of Microbes in Biofilms
Source: Front Microbiol. 2017 Sep 27;8:1865. doi: 10.3389/fmicb.2017.01865 (PMC5623813; doi:10.3389/fmicb.2017.01865)
Supplement: Supplementary file 1 [file Presentation1.PDF]

Supporting information

Extracellular polymeric substance production and aggregated bacteria colonization influence the competition of microbes in biofilms

Pahala Gedara Jayathilake[1], Saikat Jana[1], Steve Rushton[2], David Swailes[3], Ben Bridgens[1], Tom Curtis[4], Jinju Chen[1]

[1]School of Engineering, Newcastle University, Newcastle upon Tyne, NE17RU, UK, [2]School of Natural and Environmental Sciences, Newcastle University, Newcastle upon Tyne, NE17RU, UK, [3]School of Mathematics, Statistics and Physics, Newcastle University, Newcastle upon Tyne, NE17RU, UK, [4]Centre for Synthetic Biology and the Bioeconomy, Newcastle University, Newcastle upon Tyne, NE24AX

Initial colonization

Three different initial colonization scenarios are as below:

1. Control: Both strains (EPS producers and non-producers) deposit on the substratum as single cells.
2. Case 1: EPS producing strain deposit as aggregates of different sizes and the non-producing strain deposit as single cells
3. Case 2: EPS producing strain deposit as single cells and the non-producing strain deposit as aggregates of different sizes.

For all the scenarios of colonization the initial cell number of each strain (EPS producers and non-producers) is 50.

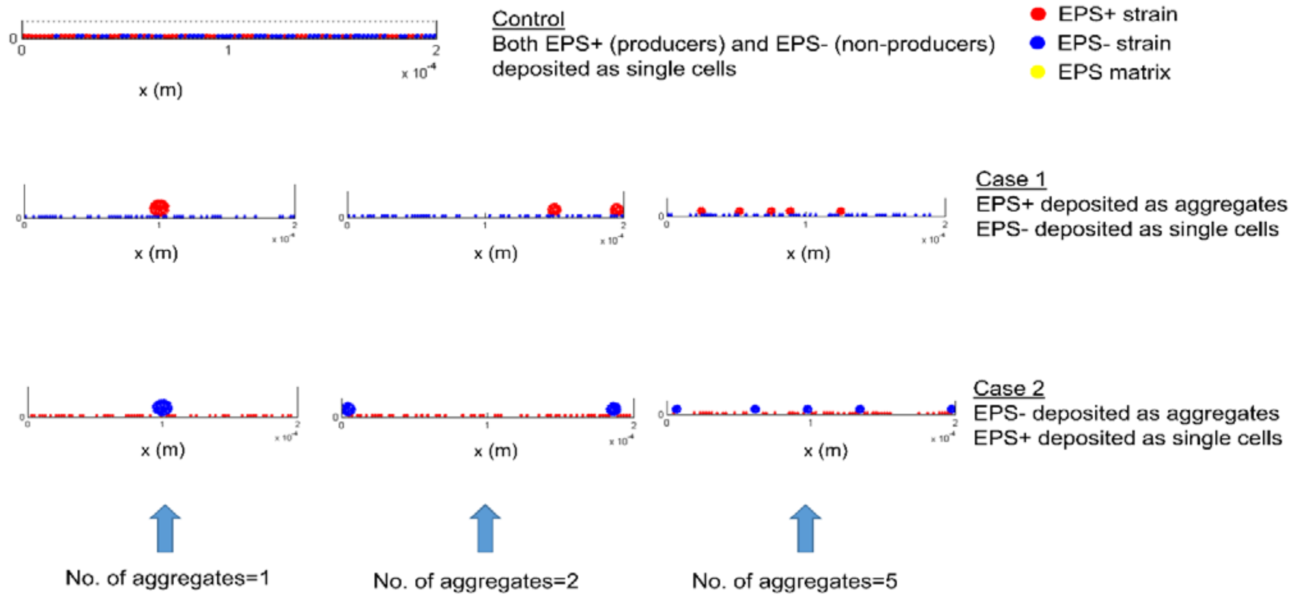

Figure S1: Initial colonization of EPS producers and non-producers. As the number of aggregates increases the size of each aggregate decreases because the initial number of cells for each strain is 50.

The results shown in Figure 6 are also presented in Figure S2 to visualize the effect of number of aggregates on the relative fitness of EPS+ strain for each case.

A

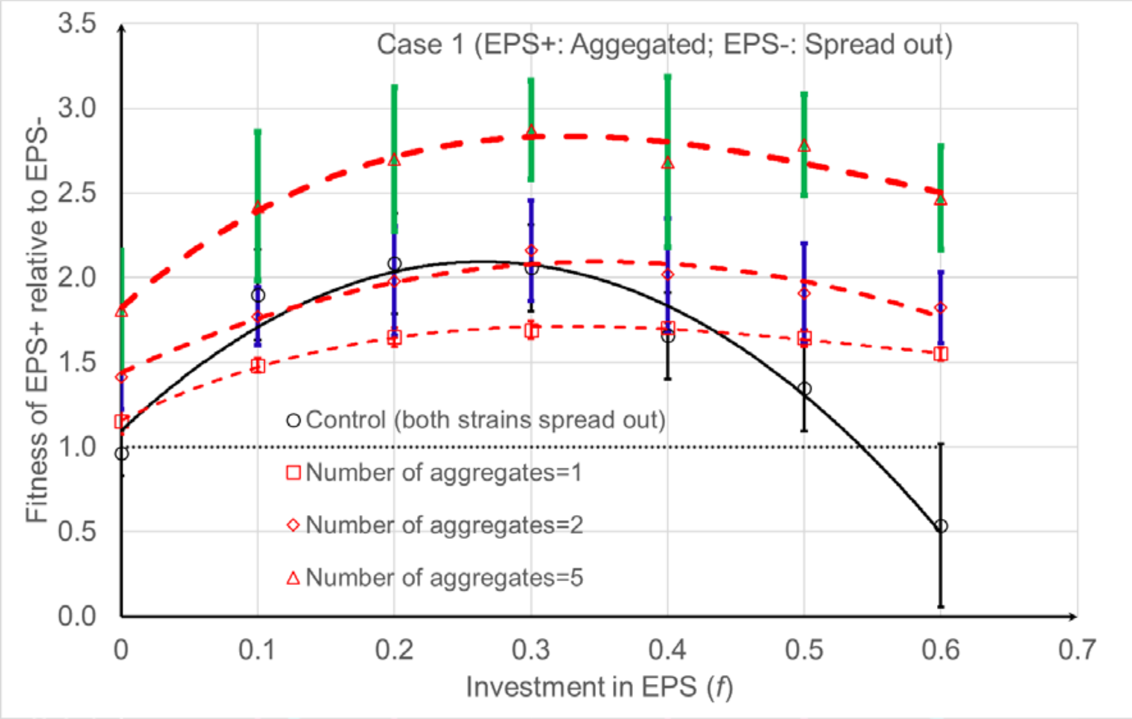

B

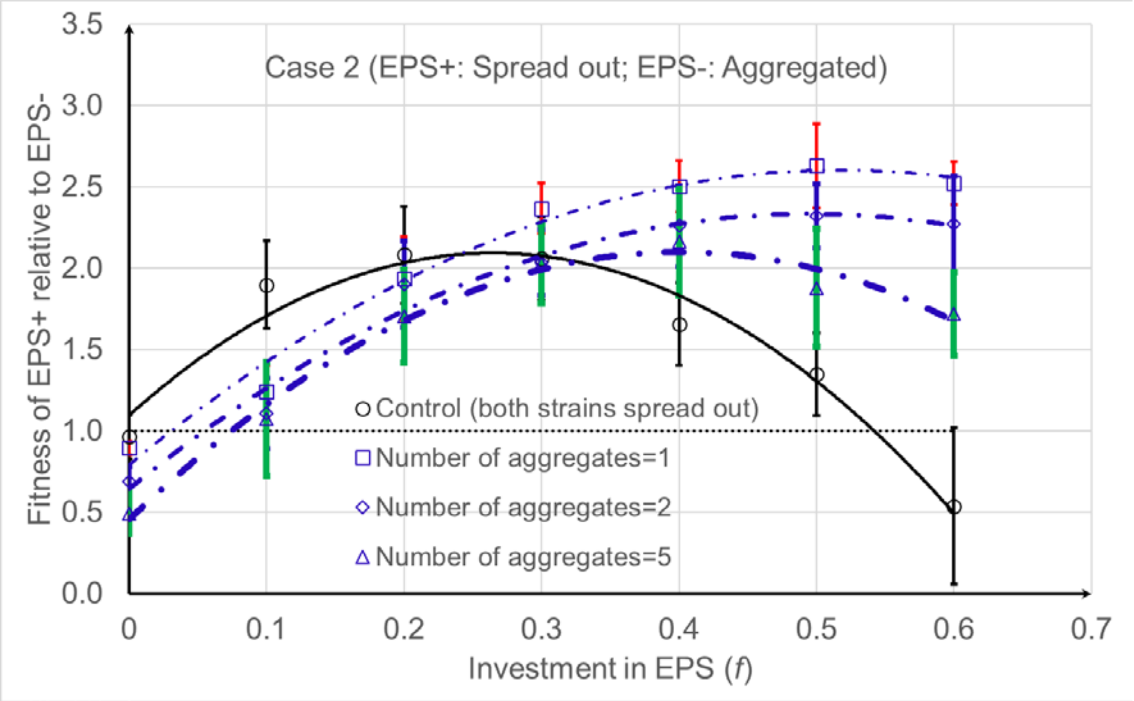

Figure S2: Effect of number of aggregates and investment in EPS: (A) Case 1, EPS+ strain deposits as aggregates and EPS- strain deposits as single cells; (B) Case 2, EPS+ strain deposits as single cells and EPS+ strain deposits as aggregates. As the number of aggregates increases (size of each aggregate decreases) the aggregated strain is benefitted by the smaller aggregates.

Generalised linear modelling (GLM)

(i) Effect of colonization scenario and aggregate size

The impacts of varying EPS investment and spatial disposition of starter population on competition between the two strains was further investigated using generalised linear modelling in and with information-theoretic model selection. A full model defining hypothesised relationships between the relative fitness of EPS+ strain and the independent variables (investment in EPS, number of aggregates and three deposition scenario) and putative interactions was used as a base model, from which the optimal model was identified through automated model selection. Models were fitted in the MuMIn package in R and compared on the basis of AIC (Akaike Information Criterion).

The relative fitness response variable was unimodal throughout all runs, indicating that there is an optimum investment level in EPS which above investment in EPS leads to a decrease in the relative fitness of EPS+ strain. We included a quadratic term for the investment in EPS (*f*) in the model to account for this behaviour. The best model included the level of EPS investment (*f*) and a quadratic term for it, the number of aggregates and three initial deposition scenarios (control, Case1, Case2) and interaction terms of these variables with *f* and its quadratic transform. This model explained approximately 63% of the variation in the original relative fitness data.

The relative fitness of EPS+ strain to EPS- strain was significantly related to the level of investment in EPS (*f*) by the EPS+ bacteria and the quadratic transform ($t=22.653$, $P < 2\times10^{-16}$ and $t=-18.486$, $P< 2\times10^{-16}$, respectively). In addition, Case 1 with higher numbers of aggregates had higher relative fitness for EPS+ strain than either control or Case 2 ($t=9.737$, $P < 2\times10^{-16}$). This indicates that EPS+ aggregates that were spread out more widely across the substratum relative to the non-EPS producers had a fitness advantage than when they were clumped into one colony relative to non-EPS producers or when they were both spread out as single cells on the surface.

There was also a significant three-way interaction between the quadratic term for EPS investment (*f*), the number of initial aggregates and the cell deposition scenario (control, Case1,

Case2). With EPS+ relative fitness being lowest ($t=-9.783$, $P< 2\times10^{-16}$) when EPS+ and EPS- strains equivalently spread out (control), were compared with EPS+ strain which are aggregated and with greater size ($t=6.315$, $P=5.15\times10^{-10}$) . Since the quadratic term effectively models the rate at which the optimum level for fitness is approached given increasing EPS investment, this means that the optimum EPS investment to maximise fitness was clearly dependent on the spread and size of aggregates in the initial population.

(ii) Effect of quorum sensing

We used generalised linear modelling to investigate the collective effects of aggregate type, quorum sensing threshold and the occurrence of up or down regulation on the relative fitness of EPS producers compared to non-producers. This model assumes that there is a linear relationship between the fitness outcome and the input variables and that the deviations (errors) associated with the model were normally distributed with zero mean. We tested these assumptions using normality plots for the residuals of the fitted model and quantified the contribution of the variables to the observed outputs from the model. We included interaction term between quorum sensing thresholds and the presence of up/down regulation of EPS production to investigate the extent to which the two variables were synergistic in their effects. The interaction term led to a better model with increased variation in output explained. The residuals from the model were normally distributed indicating that the underlying assumptions of the linear model with normal errors was adequate.

There was a very significant effect of quorum sensing threshold on relative fitness ($t=-6.540$ $P=3.83e-10$) and the occurrence of up versus down regulation of EPS production ($t=-10.248$ $< 2e-16$). There was a significant interaction between the quorum sensing threshold and whether the EPS was up regulated or down regulated ($t=13.868 < 2e-16$) indicating a synergy between the two variables in their effects on fitness. Relative fitness was also dependent on the aggregation case, with aggregated EPS producers (case 2) leading to higher fitness than other two cell deposition scenarios ($t=6.660$ $P=1.94e-10$). Together these variables explained 54% of the variation in fitness suggesting that the random nature of the demographic processes in the model were responsible for the remainder of the observed variation in fitness of EPS producers.
